# Exploring novel experimental treatments for major neurodegenerative disorders

**DOI:** 10.1002/nep3.31

**Published:** 2023-12-17

**Authors:** Xunming Ji, Piotr Walczak, Johannes Boltze

**Affiliations:** 1 Beijing Institute of Brain Disorders, Capital Medical University, Beijing, China; 2 Department of Diagnostic Radiology and Nuclear Medicine, Center for Advanced Imaging Research, University of Maryland, Baltimore, Maryland, USA; 3 School of Life Sciences, University of Warwick, Coventry, UK

**Keywords:** Alzheimer's disease, amyotrophic lateral sclerosis, cell therapy, cytoprotection, extracellular vesicles, neurodegeneration, stroke

Acute and chronic neurodegenerative disorders such as ischemic stroke or Alzheimer's disease (AD) impose a major burden on patients, their relatives, caregivers, and health care systems in general. The socioeconomic impact of neurodegenerative disorders is anticipated to escalate due to a globally ageing population and the increasing prevalence of sedentary lifestyles and inappropriate dietary habits. On the contrary, there is a paucity of therapeutic options providing causative treatment for neurodegenerative disorders, effectively mitigating their consequences and enhancing patients' quality of life. The few therapeutic options being available are often limited by temporal restrictions. For instance, recanalization approaches for ischemic stroke have a narrow therapeutic time window, rendering them inaccesible for the majority of patients. Current work therefore focuses on improving acute stroke management including adjuvant neuroprotective or immunomodulative interventions to allow more patients to benefit from recanalization.^1^ Similarly, recent approaches to target amyloid β (Aβ) plaques using monoclonal antibodies in AD are only effective in the early stages of the disease. However, the overall therapeutic impact remains modest, accompanied by the potential of severe side effects.^2^

Hence, there is a pressing need for novel therapeutic approaches. This issue of *Neuroprotection* features some exciting updates on experimental therapeutic research for neurodegenerative diseases, complemented by meta-analyses and reviews of previous, current, and future approaches. This editorial will summarize and highlight the most important aspects presented within these contributions.

Liu et al. suggest an orchestrated, global strategy to combat AD, through the AD Neuroprotection Research Initiative (ADNRI). In their comprehensive assessment of current therapeutic opportunities for AD, they stress the importance of neuroimaging approaches at both cellular and system levels for clinical disease monitoring and translational activities. They also discuss current as well as potential future approaches for neuroprotective interventions in AD, providing a detailed overview of neurotrophic factors and anti-inflammatory treatments. They further highlight the role of astrocytes and oligodendrocytes in AD. They finally focus on metabolic, vascular, and meningeal aspects of AD, as well as potential peripheral interventions and lifestyle changes, thus offering a holistic picture of therapeutic opportunities.

The work by Taguchi et al. interconnects with the insights offered by Liu and colleagues. The modest impact of anti-Aβ immunotherapies^3^ indirectly suggests that Aβ may not singularily constitute the entire pathomechanism in AD and consequently, may not be the only therapeutic target. The authors advocate for exploring potential targets such as the hippocampal neurogenesis as a viable avenue. Their rationale is based on the noteable decline in hippocampal neurogenesis among AD patients.^4^ Taguchi et al. not only provide a detailed rationale for their suggestion but also highlight the need for combination treatments to counter the complex AD pathology. They also claim that cell-based interventions hold promise for improving hippocampal neurogenesis.

Cell-based interventions for hypoxic–ischemic encephalopathy (HIE) in newborns are investigated by Scrutton and colleagues. Performing a systematic literature review and meta-analysis, they compared the therapeutic impact of mononuclear cells (MNCs) in preclinical versus clinical studies in HIE. MNCs are a stem cell-containing population that can be obtained from umbilical cord blood or bone marrow and that is commonly used in early-stage clinical investigations due to their excellent safety profile and the use for swift, autologous application. There were no strong indicators for MNC efficacy in the early clinical studies included in the analysis, but clear indications for major design differences between preclinical and clinical trials. Interestingly, such variations in design have been hypothesized to contribute to the limited clinical therapeutic impact of cell treatments for stroke.^5^ One major difference identified was the absence of therapeutic hypothermia in preclinical studies focused on HIE. This is surprising because therapeutic hypothermia is established as an HIE treatment in clinical routine and thus should be considered in preclinical studies as well.

Additional perspectives on cell therapies and their potential role as adjuvant treatments are provided by Bakreen and colleagues. Focusing on ischemic stroke, the authors describe the impact of combining experimental neurorehabilitation with cell therapies. They suggest that both intracerebral and systemic cell treatments have the potential to augment the effects of experimental neurorehabilitation. The paper also investigates the impact of the cell transplantation route and cell transplant “priming” for better integration and outcome. It further discusses the impact of cell therapies on specific neurorehabilitation paradigms. Bakreen and colleagues then suggest options to maximize the therapeutic impact of combined neurorehabilitation and cell treatments; for instance, by mimicking patient populations with specific characteristics in preclinical studies for a more focused and hopefully successful clinical translation.

Experimental approaches for improving functional recovery after stroke are also discussed by Waseem and colleagues, with a distinct focus on extracellular vesicles (EVs, also referred to as exosomes) being secreted by numerous stem cell populations. Mesenchymal stem cell (MSC)-derived EVs are of primary interest in the field as they can be obtained in large quantities, at reproducible quality, and are easy to store. The review by Waseem and colleagues provides a comprehensive overview of the benefits associated with MSC-derived EVs over MSCs and explains concepts on their potential modes of action including anti-inflammatory, antioxidative, and antiapoptotic effects. The authors further investigate the intriguing role of EVs as diagnostic biomarkers, adding depth to our understanding of their multifaceted work. The review cumulates in a discerning of existing knowledge gaps, paving the way for future research directions in this evolving field.

The advantages of EVs in experimental neurology are further stressed in the review of Lockard et al., with a specific focus on amyotrophic lateral sclerosis (ALS). Building on promising findings from experimental cell-based interventions in ALS, the authors describe the effects of EVs derived from different sources in ALS animal models. Special attention is paid to the anti-inflammatory effects of EVs and their potential role as biomarkers in ALS, drawing connections to recent clinical observations. Thus, this paper complements the work of Waseem and colleagues.

Finally, Talhada et al. report the results of an experimental study investigating if and how dopamine signaling modulates GABAergic neurotransmission and functional recovery after experimental ischemic stroke. They found that dopamine D2 receptors mediate beneficial effects after stroke and that reduced expression of cytoplasmic glutamate decarboxylase 67 (GAD 67) as well as increased cleavage of GAD 65 may be potential modes of action, explaining a direct impact of dopamine signaling on GABAergic neurotransmission. This may provide a novel therapeutic anchor point helping to improve functional outcome after ischemic stroke.

The issue also contains a clinical case study not directly related to neurodegenerative disorders, but makes an interesting contribution to the scientific discussion of long-term complications of coronavirus disease (COVID) infections. Mendoza-Portillo et al. describe jugular vein thrombosis causing neurological symptoms in a 41-year-old patient. Since that condition manifested within a day after receiving the first dose of an adenovirus-based anticoronavirus vaccination, the authors first presumed a rare complication in which vaccination-induced antibodies crossreact with platelet epitopes, mimicking coronavirus surface antigens. This may lead to blood clotting and vaccine-induced immune thrombotic thrombocytopenia. However, additional investigations did not confirm this initial assumption, and the reported events were therefore considered to be more likely a long-term COVID sequel. Although there was a mild cognitive impairment in the early stages after the event, the patient fully recuperated eventually as confirmed by a 10-week follow-up.

In summary, the current issue of *Neuroprotection* gives intriguing insights into potential novel treatment approaches at multiple mechanistic levels, along with a clinical perspective on the hot contemporary topic of long-term COVID sequelae.
